# Single-session communication with a locked-in patient by functional near-infrared spectroscopy

**DOI:** 10.1117/1.NPh.4.4.040501

**Published:** 2017-12-23

**Authors:** Androu Abdalmalak, Daniel Milej, Loretta Norton, Derek B. Debicki, Teneille Gofton, Mamadou Diop, Adrian M. Owen, Keith St. Lawrence

**Affiliations:** a Lawson Health Research Institute, Imaging Program, London, Ontario, Canada; b Western University, Department of Medical Biophysics, London, Ontario, Canada; c Western University, Brain and Mind Institute, London, Ontario, Canada; d Western University, Clinical Neurological Sciences, London, Ontario, Canada

**Keywords:** functional near-infrared spectroscopy, time-resolved measurements, Guillain–Barré syndrome, motor imagery, brain–computer interface

## Abstract

There is a growing interest in the possibility of using functional neuroimaging techniques to aid in detecting covert awareness in patients who are thought to be suffering from a disorder of consciousness. Immerging optical techniques such as time-resolved functional near-infrared spectroscopy (TR-fNIRS) are ideal for such applications due to their low-cost, portability, and enhanced sensitivity to brain activity. The aim of this case study was to investigate for the first time the ability of TR-fNIRS to detect command driven motor imagery (MI) activity in a functionally locked-in patient suffering from Guillain–Barré syndrome. In addition, the utility of using TR-fNIRS as a brain–computer interface (BCI) was also assessed by instructing the patient to perform an MI task as affirmation to three questions: (1) confirming his last name, (2) if he was in pain, and (3) if he felt safe. At the time of this study, the patient had regained limited eye movement, which provided an opportunity to accurately validate a BCI after the fNIRS study was completed. Comparing the two sets of responses showed that fNIRS provided the correct answers to all of the questions. These promising results demonstrate for the first time the potential of using an MI paradigm in combination with fNIRS to communicate with functionally locked-in patients without the need for prior training.

## 1 Introduction

Disorders of consciousness (DOC) are conditions in which normal consciousness is impaired as a result of brain damage. These disorders are classified based on a patient’s level of arousal and awareness, with vegetative state (VS) patients only exhibiting evidence of arousal and minimally conscious state (MCS) patients displaying inconsistent signs of awareness.^1^ The difficulties of differentiating between these states using behavioral tests are reflected in the high rate of MCS patients being misdiagnosed as being vegetative (up to 40%).^2^ One approach for improving differential diagnosis is to use functional neuroimaging to detect activation in specific brain regions in response to command-following tasks. This was first demonstrated using functional magnetic resonance imaging (fMRI) to detect motor imagery (MI) activity in a patient diagnosed as being vegetative.^3^

Given the limitations associated with fMRI in terms of cost and accessibility, there is an unmet need to develop techniques to detect command-driven brain activity at the bedside. Not only would this help differentiate between VS and MCS, such techniques could also provide a rudimentary means of communicating with DOC patients.^3^^–^^5^ Functional near-infrared spectroscopy (fNIRS) is a promising alternative to fMRI given its portability and relatively low cost; however, despite these advantages, only one fNIRS study to date attempted to use an MI paradigm to assess residual brain function in DOC patients and significant effects were only found at the group level.^6^ Although promising, assessing consciousness in DOC patients requires a method that can reliability detect activation on a single-subject basis.

One of the challenges with fNIRS is its inherent sensitivity to light absorption in superficial tissue, which can reduce the reliability of detecting brain activity. One approach for enhancing depth sensitivity is to use time-resolved (TR) NIRS.^7^^,^^8^ Depth sensitivity is achieved by discriminating between early arriving photons that only interrogate the extracerebral layers and late-arriving photons that have a higher probability of reaching the brain.^9^^,^^10^ Previous work has shown that TR-NIRS provides a higher contrast-to-noise ratio (CNR) compared to conventional continuous wave (CW) approaches.^11^ In addition, in a recent study involving healthy participants performing MI, it was demonstrated that the change in the mean time-of-flight (⟨t⟩) signals could be used to detect MI activation with a sensitivity of 93% compared to fMRI, whereas the change in the number of photons, which is analogous to the CW approaches, only provided a sensitivity of 64%.^12^ Given these promising results, the first aim of this case study was to investigate if the same TR-fNIRS approach could detect MI activity in a locked-in patient under intensive care. This patient had Guillain–Barré syndrome (GBS), an acute paralytic neuropathy,^13^ that in severe cases results in a functionally locked-in state. The second aim was to determine if the patient without any prior training could use MI to respond to a series of yes/no questions. In general, locked-in patients represent an ideal case to test the method in the intensive-care unit (ICU) given they are awake and aware, unlike DOC patients but lack almost all ability to respond to commands.^14^ At the time of this experiment, the patient had regained limited eye movement, which provided a unique opportunity to confirm the answers obtained by fNIRS.

## 2 Methods

This study was conducted on a patient with severe GBS who required ventilator support and was under intensive care at London Health Science Centre (London, Ontario). The patient (male, age 75, Hughes GBS disability scale score of 5 on a scale where 0 indicates normal and 6 corresponds to death) was functionally locked-in with no voluntary control of his muscles except for very restricted (few millimeters) vertical and horizontal eye movements, which were inconsistent in the days leading up to this study. Prior to becoming functionally locked-in, the patient requested to remain unsedated once in a locked-in state. His decision allowed us to test our MI approach on a patient completely free of the effects of sedatives. This study was approved by the Research Ethics Board of Western University and informed consent was obtained from the patient’s legal guardian.

**Fig. 1:**
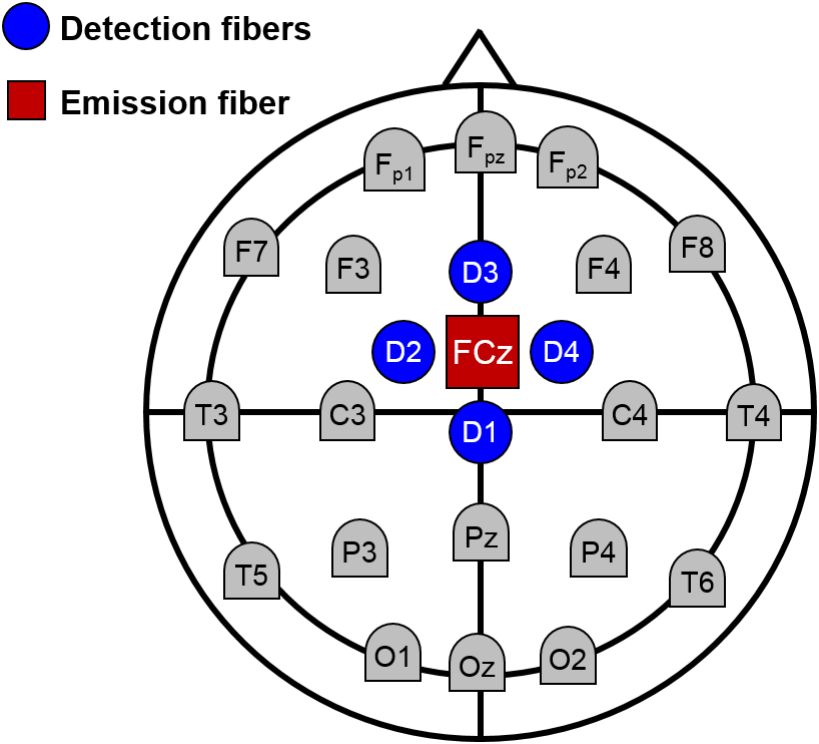
Schematic of the TR-fNIRS probes on the head. The red circle illustrates the location of the emission fiber (FCz), whereas the blue circles represent the detection fiber positions with a source–detector distance of 3 cm.

The fNIRS data were acquired with a four-channel TR system described in details elsewhere.^12^^,^^15^ Briefly, the system is optimized to detect activation in the motor planning regions: the supplementary motor area (SMA) and the premotor cortex (PMC).^12^ A bifurcated emission fiber was centered over FCz according to the international system for electroencephalography (EEG) electrode placement, and the detection fiber bundles were placed in a cross orientation with a source–detector distance of 3 cm (Fig. 1). The fibers were secured to the head using a three-dimensional printed holder imbedded in an EEG cap (EASYCAP, GmbH, Germany). Ultrashort pulses of light were emitted at 760 and 830 nm and at a pulse repetition rate of 80 MHz. Distribution of time-of-flight of photons (DTOFs) were acquired every 300 ms with a temporal resolution of 16 ps. The system was controlled using custom LabVIEW software (National Instruments).

**Fig. 2:**
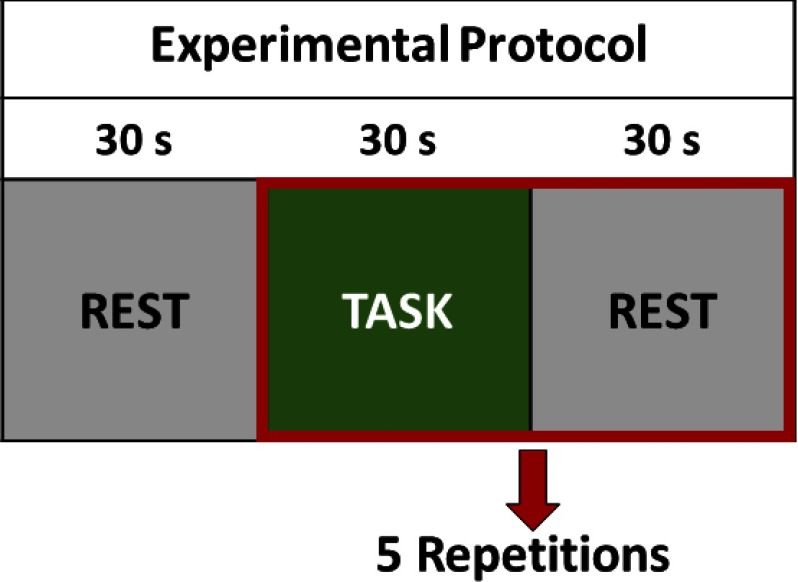
Schematic of the MI paradigm used to communicate with the patient. Following 30 s of rest period, each question was repeated five times in a alternating block design of task and rest period for a total experiment time of 5:30 minutes per question. During the task period, the patient was instructed to imagine playing tennis if he wanted to answer “yes” or to stay relaxed if he wanted to answer “no.”

MI was invoked using a well-established “imagine playing tennis” task that required subjects to imagine themselves on a tennis court playing a vigorous game of tennis where they are swinging their arm back and forth trying to hit a tennis ball over and over.^3^^,^^16^ The fNIRS experiment was organized in two parts: first, the patient was instructed to perform the tennis imagery task to verify his ability to successfully perform MI. The experimental protocol consisted of 30 s of rest followed by five 30-s alternating blocks of MI and rest for a duration of 330 s. Next, the patient was asked three questions confirming his last name, if he was in pain, and if he felt safe. The first question was chosen as a control, whereas the other two open-ended questions were chosen for their clinical relevance. He was instructed to stay relaxed if he wanted to answer “no” to any of the questions or to perform tennis imagery if the answer was “yes.” Each question was repeated five times in the same block design of 30-s intervals used for the MI task.^12^^,^^16^ A schematic of the paradigm is presented in Fig. 2. Immediately following the fNIRS experiment, the patient answered the same three questions using vertical (“yes”) and horizontal (“no”) eye movements while his eyelids were held open.

The fNIRS signals were analyzed by calculating the change in the statistical moments of each recorded DTOF.^7^ Only the change in the ⟨t⟩ was used in the analysis since the previous study demonstrated that it provided the highest sensitivity to MI activity.^12^ All ⟨t⟩ time courses were corrected for motion artifacts using the movement reduction artifact rejection algorithm approach,^17^ filtered using a band-stop filter with cut-off frequencies of 0.08 and 1.5 Hz, and detrended to remove any slow signal drifts.^12^ Finally, the signals were converted to oxy- and deoxyhemoglobin using sensitivity factors (SF) obtained from Monte Carlo simulations.^18^ The Monte Carlo model consisted of 10 layers, each with a thickness of 0.2 cm. The SF for the intracerebral layer was calculated as the sum of the SF obtained from layers 5 to 10 (i.e., below 1 cm).

An increase in oxyhemoglobin during MI from each of the four detection channels was detected by a support vector machine classifier. The CNR and the correlation coefficient (r) between the oxyhemoglobin time course and the theoretical model were used as features to train the classifier. The CNR was defined as the difference in signal between the mean task and rest periods divided by the standard deviation of the rest period. The classifier was trained on one hundred simulated data sets with varying degrees of noise added to replicate experimental data. The theoretical activation signals were simulated as a five-cycle boxcar convolved with the hemodynamic function, whereas the rest signals were simulated as the combination of three sinusoidal signals with frequencies of 0.1, 0.2, and 1 Hz corresponding to the Mayer waves, average breathing rate, and average heart rate, respectively. Random noise was added with a normal distribution and standard deviation ranging from 1 to 10.

Testing on fifteen MI and five rest data collected previously from healthy controls^12^ demonstrated that the classifier had an accuracy of 80% and a precision of 75%. The final step was to confirm the “yes” or “no” responses obtained by applying the classifier to the oxyhemoglobin time series by comparison to the responses obtained by eye movements. Since all four channels were located over motor planning regions, at least one of them had to be classified as activated for a “yes” response.

## 3 Results

**Fig. 3:**
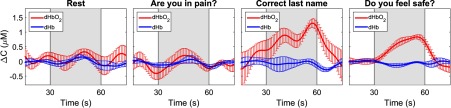
Changes in the concentration of oxyhemoglobin (red) and deoxyhemoglobin (blue) averaged across all five cycles for each of the three questions. For the responses classified as “yes” (i.e., correct last name and do you feel safe), the signals were averaged across all activated channels, whereas for the response classified as “no” (i.e., are you in pain), the signals were averaged across all four channels. The baseline time course labeled “rest” refers to data acquired without MI activation and is presented as a reference for the contrast observed during the question periods. The error bars represent the standard error of mean across the specific activated/inactivated channels. The gray boxes indicate the response period.

Analysis of the fNIRS data acquired during tennis imagery alone revealed activation in one channel. Compared to the responses using eye movements, the fNIRS results predicted the correct answers to all three questions: (1) “yes,” the patient heard his last name (three channels, average CNR=5.85, average r=0.76); (2) “no,” he was not in pain (four channels, average CNR=1.13, average r=0.23); and (3) “yes,” he felt safe (four channels, average CNR=12.72, average r=0.83). The CNR and correlation values for the “yes” responses were similar to that of healthy participants performing the same MI task.^12^ The average time courses of oxy- and deoxyhemoglobin concentrations for each of the three questions are shown in Fig. 3.

## 4 Discussion

The main result of this case study was to demonstrate that a four-channel TR-fNIRS system could detect command-driven brain activity in a functionally locked-in patient in the ICU. By implementing an MI paradigm validated in healthy controls and by strategically targeting motor-planning regions, rudimentary communication was conducted with a patient who had undergone no previous training. A major strength of this study was confirmation of the fNIRS results since the patient was able to answer the same questions with eye movements.

The MI trial performed at the beginning of this study confirmed the patient’s ability to perform the task, which is essential if a patient is going to use MI to respond to questions. Compared with the data from the two “yes” responses in which MI activity was detected in at least three channels, the MI trial only resulted in detectable activity in a single channel. It could be expected that the number of probes detecting MI activity should be fairly consistent across trials. In this experiment, the positions of the four probes were adjusted after the MI task, due to evidence of suboptimal signals, which could explain the difference in activated channels between trials.

As a feasibility study, the number of questions was limited to three due to time constraints in the ICU (each question was repeated five times for a total of 5:30 min per question). An additional concern was the potential for patient fatigue as roughly a third of GBS patient exhibits mental status abnormalities.^19^ Consequently, priority was given to clinically relevant questions instead of including an incorrect autobiographical question (e.g., false name) to demonstrate that the method can accurately predict a correct “no” response. However, the negative response to the question: “are you in pain” obtained by both communication methods demonstrated the ability of the fNIRS approach to confirm a negative response. In order to reduce the overall time per question, further testing is required to determine how many cycles are required to obtain a reliable answer. For this case study, a five-cycle MI protocol was adopted since it has been rigorously tested in both fMRI and fNIRS studies.^3^^,^^12^

A potential limitation with the fNIRS method used in this study was the lack of a task-driven “no” response, which raises uncertainties as to whether or not a lack of MI activity truly indicates “no” or just a lack of awareness. To address this issue, the patient was first asked to perform MI prior to using this mental activity to answer questions. However, a “no” response involving another mental imagery task that activates different brain regions, such as spatial navigation,^16^ could enhance the confidence in negative answers since it would elicit its own activation pattern. The current method could be extended to monitor another brain region, such as regions of the parietal cortex associated with spatial navigation, but this should be validated in control studies prior to translation to DOC patients. Another frequent issue with fNIRS studies is the potential for signal contamination from changes in systemic physiology,^20^^,^^21^ particularly heart rate and arterial ${\mathrm{CO}}_{2}$ tension caused by changes in respiration. In this case, the patient’s heart rate was monitored and no changes were observed during the task periods. Furthermore, the patient was mechanically ventilated so there were no changes in respiration rate.

The potential of using fNIRS as a BCI to communicate with locked-in patients was recently demonstrated by Chaudhary et al.^22^ and Gallegos-Ayala et al.^23^ In these studies, each patient underwent a battery of training sessions in order to establish individual “yes” and “no” fNIRS responses. This approach is intended for patient populations who lack any physical ability to communicate but retain full awareness, such as those with late-stage amyotrophic lateral sclerosis. This is different from the approach used in this current study that requires participants to perform a specific mental imagery task and was originally designed to assess awareness by detecting command-following activation. Although in this study MI was used for rudimentary communication, the primary goal is to develop an fNIRS technique that can reliably assess consciousness at the bedside of DOC patients.

Finally, translating this research to DOC patients may pose certain technical challenges. First, brain damage in DOC patients could result in postinjury brain reorganization. This would affect the choice of probe placement if the position of the SMA and PMC relative to the 10-10 EEG template was altered. Furthermore, some patients may also suffer from brain ischemia or hematoma, which can affect the scattering and absorption of light, respectively. It will likely be crucial to examine each patient’s imaging data, either computed tomography (CT) or MRI, prior to applying the fNIRS BCI method. For patients who have undergone previous fMRI scans and who do not suffer from damage to the secondary motor regions of the brain, their scans could be used to guide probe placement on the scalp. On the other hand, for patients with damage to the SMA and/or PMC, alternate paradigms that activate other cortical regions, such as spatial navigation, could be used. Finally, while detecting MI activity in DOC patients can be used to infer covert awareness, no claims about residual awareness can be made from a negative finding (i.e., failing to detect MI activity). As a result, conclusions regarding the preservation of awareness in DOC patients should be drawn from positive outcomes only.^16^

In summary, this case study demonstrated the potential of using fNIRS as a bedside tool to detect command-driven brain activity in an ICU patient who had extremely limited physical ability to communicate. The results suggest that fNIRS could be used to ask patients questions that have a direct bearing on their clinical management, particularly regarding pain and other aspects of well-being. To our knowledge, this is the first account of an fNIRS approach being used to communicate with a locked-in patient without the need for prior training. The accuracy of the approach was confirmed by obtaining ground truth answers through eye movements. Given the portability of fNIRS, repeat measurements could be performed to monitor levels of awareness and perhaps assist in patient prognosis. Future work will focus on testing the approach on a larger cohort of locked-in and DOC patients to estimate reliability and reproducibility

## References

[1] Laureys S. Owen A. M. Schiff N. D., “Brain function in coma, vegetative state, and related disorders,” Lancet Neurol. 3, 537–546 (2004). 10.1016/S1474-4422(04)00852-X 15324722

[2] Schnakers C. et al., “Diagnostic accuracy of the vegetative and minimally conscious state: clinical consensus versus standardized neurobehavioral assessment,” BMC Neurol. 9, 35 (2009). 10.1186/1471-2377-9-35 19622138 PMC2718857

[3] Owen A. M. et al., “Detecting awareness in the vegetative state,” Science 313(5792), 1402–1402 (2006). SCIEAS 0036-8075 10.1126/science.1130197 16959998

[r4] Monti M. M. et al., “Willful modulation of brain activity in disorders of consciousness,” N. Engl. J. Med. 362, 579–589 (2010). NEJMAG 0028-4793 10.1056/NEJMoa0905370 20130250

[5] Cruse D. et al., “Bedside detection of awareness in the vegetative state: a cohort study,” Lancet 378, 2088–2094 (2011). LANCAO 0140-6736 10.1016/S0140-6736(11)61224-5 22078855

[6] Kempny A. M. et al., “Functional near infrared spectroscopy as a probe of brain function in people with prolonged disorders of consciousness,” NeuroImage Clin. 12, 312–319 (2016). 10.1016/j.nicl.2016.07.013 27547728 PMC4983150

[7] Liebert A. et al., “Time-resolved multidistance near-infrared spectroscopy of the adult head: intracerebral and extracerebral absorption changes from moments of distribution of times of flight of photons,” Appl. Opt. 43, 3037–3047 (2004). APOPAI 0003-6935 10.1364/AO.43.003037 15176190

[8] Farina A. et al., “In-vivo multilaboratory investigation of the optical properties of the human head,” Biomed. Opt. Express 6(7), 2609–2623 (2015). BOEICL 2156-7085 10.1364/BOE.6.002609 26203385 PMC4505713

[9] Milej D. et al., “Optimization of the method for assessment of brain perfusion in humans using contrast-enhanced reflectometry: multidistance time-resolved measurements,” J. Biomed. Opt. 20(10), 106013 (2015). JBOPFO 1083-3668 10.1117/1.JBO.20.10.106013 26509415

[10] Milej D. et al., “Time-resolved subtraction method for measuring optical properties of turbid media,” Appl. Opt. 55(7), 1507–1513 (2016). APOPAI 0003-6935 10.1364/AO.55.001507 26974605

[11] Selb J. et al., “Improved sensitivity to cerebral hemodynamics during brain activation with a time-gated optical system: analytical model and experimental validation,” J. Biomed. Opt. 10(1), 011013 (2005). JBOPFO 1083-3668 10.1117/1.1852553 15847579

[12] Abdalmalak A. et al., “Can time-resolved NIRS provide the sensitivity to detect brain activity during motor imagery consistently?” Biomed. Opt. Express 8(4), 2162–2172 (2017). BOEICL 2156-7085 10.1364/BOE.8.002162 28736662 PMC5516814

[13] Willison H. J. Jacobs B. C. van Doorn P. A., “Guillain–Barré syndrome,” Lancet 388(10045), 717–727 (2016). LANCAO 0140-6736 10.1016/S0140-6736(16)00339-1 26948435

[14] Lulé D. et al., “Life can be worth living in locked-in syndrome,” Prog. Brain Res. 177, 339–351 (2009). PBRRA4 0079-6123 10.1016/S0079-6123(09)17723-3 19818912

[15] Abdalmalak A. et al., “Assessing the feasibility of time-resolved fNIRS to detect brain activity during motor imagery,” Proc. SPIE 9690, 969002 (2016). PSISDG 0277-786X 10.1117/12.2209587

[16] Fernández-Espejo D. Norton L. Owen A. M., “The clinical utility of fMRI for identifying covert awareness in the vegetative state: a comparison of sensitivity between 3T and 1.5T,” PLoS One 9(4), e95082 (2014). POLNCL 1932-6203 10.1371/journal.pone.0095082 24733575 PMC3986373

[17] Scholkmann F. et al., “How to detect and reduce movement artifacts in near-infrared imaging using moving standard deviation and spline interpolation,” Physiol. Meas. 31(5), 649–662 (2010). PMEAE3 0967-3334 10.1088/0967-3334/31/5/004 20308772

[18] Milej D. et al., “Subtraction-based approach for enhancing the depth sensitivity of time-resolved NIRS,” Biomed. Opt. Express 7(11), 4514–4526 (2016). BOEICL 2156-7085 10.1364/BOE.7.004514 27895992 PMC5119592

[19] Cochen V. et al., “Vivid dreams, hallucinations, psychosis and REM sleep in Guillain–Barré syndrome,” Brain 128(11), 2535–2545 (2005). BRAIAK 0006-8950 10.1093/brain/awh585 16000335

[20] Tachtsidis I. Scholkmann F., “False positives and false negatives in functional near-infrared spectroscopy: issues, challenges, and the way forward,” Neurophotonics 3(3), 031405 (2016). 10.1117/1.NPh.3.3.031405 27054143 PMC4791590

[21] Kirilina E. et al., “The physiological origin of task-evoked systemic artefacts in functional near infrared spectroscopy,” Neuroimage 61(1), 70–81 (2012). NEIMEF 1053-8119 10.1016/j.neuroimage.2012.02.074 22426347 PMC3348501

[22] Chaudhary U. et al., “Brain–computer interface–based communication in the completely locked-in state,” PLoS Biol. 15(1), e1002593 (2017). 10.1371/journal.pbio.1002593 28141803 PMC5283652

[23] Gallegos-Ayala G. et al., “Brain communication in a completely locked-in patient using bedside near-infrared spectroscopy,” Neurology 82(21), 1930–1932 (2014). NEURAI 0028-3878 10.1212/WNL.0000000000000449 24789862 PMC4049706

